# Sigmoid stenosis caused by diverticulosis mimicking advanced colorectal cancer

**DOI:** 10.1093/jscr/rjae255

**Published:** 2024-04-24

**Authors:** Svetlana Shumarova, Anton Koichev, Manol Sokolov

**Affiliations:** Department of Surgery, University Hospital “Aleksandrovska” Sofia, Bulgaria, Medical University, 1 Georgi Sofijski Blvd 1431, SofiaBulgaria; Department of Surgery, University Hospital “Aleksandrovska” Sofia, Bulgaria, Medical University, 1 Georgi Sofijski Blvd 1431, SofiaBulgaria; Department of Surgery, University Hospital “Aleksandrovska” Sofia, Bulgaria, Medical University, 1 Georgi Sofijski Blvd 1431, SofiaBulgaria

**Keywords:** diverticulosis, colon cancer, stenosis

## Abstract

Stenosis is a rare complication of acute diverticulitis, difficult to differentiate from colon cancer. We present a 63-year-old woman with right lumbar pain radiating to the back. A sigmoid stenosis was detected by magnetic resonance imaging. Three biopsies were performed, all of which were negative for malignancy. From CT images with data of circumferentially thickened intestinal wall along 6 cm, stenosing the lumen enlarged regional lymph nodes. A sigmoid resection was performed and the results of histological examination showed complicated diverticulitis of the large intestine with exacerbation, abscending and spread of the inflammatory process with involvement of the pericolic tissues. Given the high risk of developing a malignant process in patients with acute diverticulitis and the slightest doubt should be followed by surgical treatment.

## Introduction

Diverticular disease affects a significant percentage of the US population with >00 000 admissions and 1.5 million hospital caries per year, with total annual hospitalizations for acute diverticulitis >7 years increasing by 26% with admission growth of 82% in ages 18 to 44 and 36% between 45 and 74 [1]. Stenosis is a rare complication of acute diverticulitis difficult to distinguish from carcinoma and with some risk of obstruction. The differentiation of benign stenosis from malignant one is extremely difficult and of essential importance given the different therapeutic behavior. A cohort study of 40 496 patients with diverticulitis found a significantly higher incidence of colon cancer in the group with diverticulitis (4.3%) compared to the group without diverticulitis (2.3%)(*P* < .001) [2]. Therefore these patients should undergo operative treatment despite the negative histological result for malignancy on endoscopic biopsy. We present a case of sigmoid stenosis caused by diverticulosis, mimicking carcinoma.

## Case report

**Figure 1:**
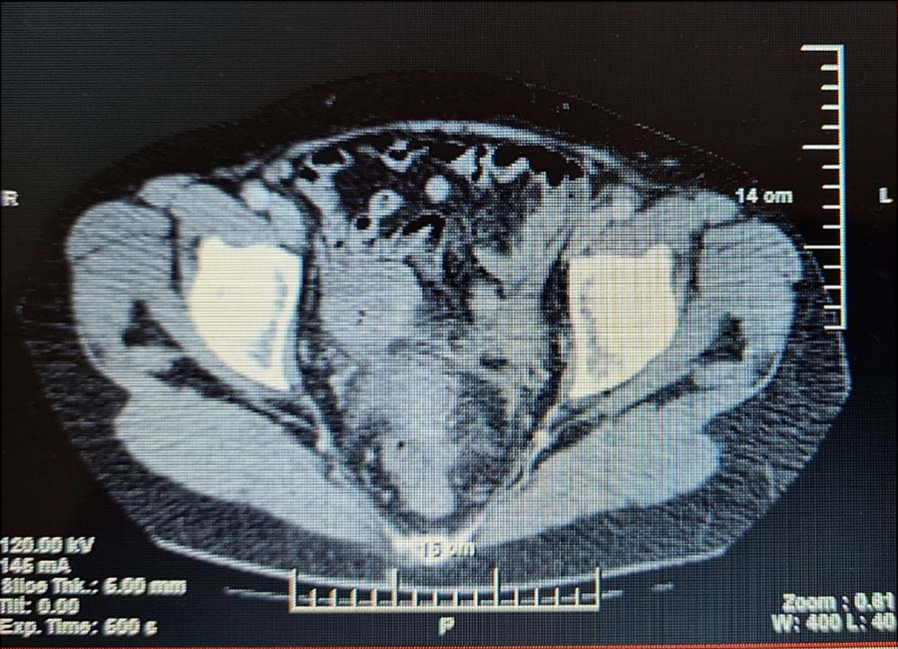
CT of the abdomen shows stenosing of the sigmoid colon.

**Figure 2:**
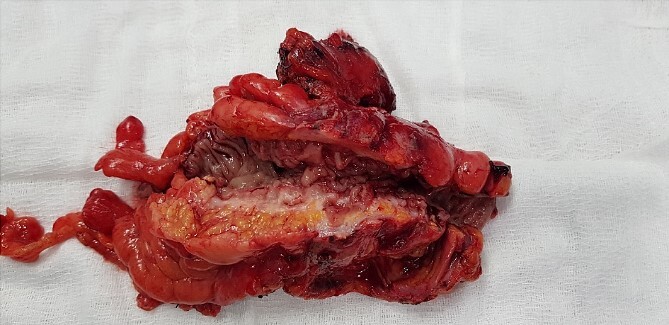
Macroscopic specimen after resection.

A 63-year- old women was admitted to the surgical department with right abdominal pain radiating to the right inguinal region and low back, and the pain had worsened in the last month. Because of these complains a magnetic resonance imaging was performed on an outpatient basis and a thickening of the sigmoid along a length of ~4 cm was found. The patient tells about a benign education, for which the necessary documentation is missing. Colonoscopy was performed which revealed edema and hyperemia of the mucosa ~20 cm from anorectal line to pseudopolypoid dilated lymphatic vessels as in a malignant process. Three biopsies were performed, the result of which proved the presence of moderately inflamed changes and marked lymphostasis with dilated lymphatics. Given the colonoscopy finding and the inability to rule out a malignant process despite a negative biopsy, a contrast- enhanced computed tomography of the abdomen and pelvis was performed. Examination showed a circumferentially and irregularly thickened intestinal bowel wall up to 15 mm ~ 11 cm from the anorectal line along ~6 cm concentrically stenosing lumen, increasing its density characteristic unevently and mainly on the periphery, compacted perirectal fatty tissue, diverticulosis along the sigmoid course, enlarged, regional lymph nodes up to 14/12 mm (Fig. 1). The blood count showed no abnormalities. Given the suspicions of the presence of a malignant process in the sigmoid, the patient underwent surgery and a thickening was found in the sigmoid area and pericolic adipose tissue with infiltration to the right ovary and right ureter. En block resection of the sigmoid along with the right ovary was performed, which was densely attached to the right ureter. On cutting a macroscopic specimen, the cartilaginous density of the tumor along a length of ~6 cm/d and stenosing the lumen of the intestine was impressed, which confirmed our suspicions of malignancy (Fig. 2). Contrary to our expectations, the histological result proved a complicated colonic diverticulitis with exacerbation, abscessation and spread of the inflammatory process with involvement of the pericolic tissues and right ovary, lymph nodes with mixed reactive lymphadenitis. The patient was followed twice with fibrocolonoscopy and with nuclear magnetic resonance postoperatively, which did not show any pathological changes.

## Discussion

The hectic lifestyle, the low culture regarding healthy nutrition are the reason why etiological factors such as low fiber content in food [3], obesity [4], smoking [5], the use of steroidal [6], and nonsteroidal [7] anti-inflammatory drugs are associated with the high incidence of diverticulitis in humans.

Very often, diverticulosis does not cause symptoms and is diagnosed when a complication occurs, as in our case. Stenosis due to fibrosis after past diverticulitis is a rare complication, often found in the presence of subileus manifastations and without history and evidence of previous episodes of diverticulitis [8]. One established, the differentiation of benign from malignant stenosis follows, which in many cases is challenging and unclear until the final histological result of the resected stenotic area is obtained, as described by Nishiyama [8] and is in the case presented by us. A retrospective study of 110 patents with complicated diverticular disease by Hussain *et al.* [9] reported the presence of phlegmon in 31% of cases and stricture/obstruction in 10%. Sigmoid colon is the most common site for complicated diverticulitis in 91% of patients [9].

A meta analysis performed by Meyer *et al.* [10] including 50 445 patients with acute diverticulitis found an overall incidence of colorectal cancer of 1.9% with a significantly higher risk in patients with complicated diverticulitis compared to patients with uncomplicated diverticulitis. A systematic review of 30 studies involving 29 348 subjects with acute diverticulitis by Kaoo *et al.* [11] found colon malignancy in 1.67% with a 1.22% risk of malignancy in patients with uncomplicated diverticulitis and 6.14% in complicated. Tehranian *et al.* [12] found no significant difference in the incidence of colorectal carcinoma in patients with complicated versus uncomplicated diverticulitis (respectively 5.6% and 7.4%), and in 69.2% the diverticulitis was diagnosed at the same site as the tumor. They concluded that colonoscopy is recommended after the diagnosis of diverticulitis, as significantly more colorectal carcinoma was detected after diverticulitis than among subjects undergoing screening colonoscopy.

Computed tomography remains the method of choice for the diagnosis of both acute diverticulitis and emerging complications with a sensitivity of 99% [13]. In the two cases describe by Nishiyama *et al*. [8] the computed tomography shows the thickening of the bowel wall, and the biopsy excludes the presence of malignancy. In the case describe by us, in addition to thickening of the wall over a length of ~6 sm/d, there are also enlarged lymph nodes, as described by Nishiyama [8] in one of the cases. Enlarged lymph nodes rather make the surgeon to think in the direction of the presence of a malignant disease, and this is a serious prerequisite for resorting to surgical treatment. Differentiating malignant from benign disease in the presence of a diverticulum near the tumor suggests complicated diverticular disease, but operative treatment and definitive histological verification are needed because a negative biopsy does not rule out colon cancer.

## Conflict of interest statement

None declared.

## Funding

None declared.
